# A Peptide That Binds Specifically to the β-Amyloid of Alzheimer's Disease: Selection and Assessment of Anti-β-Amyloid Neurotoxic Effects

**DOI:** 10.1371/journal.pone.0027649

**Published:** 2011-11-10

**Authors:** Fang Wang, Xian-Ling Zhou, Qi-Gang Yang, Wen-Hua Xu, Fei Wang, Yong-Ping Chen, Gui-Hai Chen

**Affiliations:** 1 Department of Neurology, The First Affiliated Hospital of Anhui Medical University, Hefei, People's Republic of China; 2 Department of Biomedical Engineering, Johns Hopkins University, Baltimore, Maryland, United States of America; University of Helsinki, Finland

## Abstract

The accumulation of the amyloid-β peptide (Aβ) into amyloid plaques, an essential event in Alzheimer's disease (AD) pathogenesis, has caused researchers to seek compounds that physiologically bind Aβ and modulate its aggregation and neurotoxicity. In order to develop new Aβ-specific peptides for AD, a randomized 12-mer peptide library with Aβ_1-10_ as the target was used to identify peptides in the present study. After three rounds of selection, specific phages were screened, and their binding affinities to Aβ_1-10_ were found to be highly specific. Finally, a special peptide was synthesized according to the sequences of the selected phages. In addition, the effects of the special peptide on Aβ aggregation and Aβ-mediated neurotoxicity *in vitro* and *in vivo* were assessed. The results show that the special peptide not only inhibited the aggregation of Aβ into plaques, but it also alleviated Aβ-induced PC12 cell viability and apoptosis at appropriate concentrations as assessed by the cell counting kit-8 assay and propidium iodide staining. Moreover, the special peptide exhibited a protective effect against Aβ-induced learning and memory deficits in rats, as determined by the Morris water maze task. In conclusion, we selected a peptide that specifically binds Aβ_1-10_ and can modulate Aβ aggregation and Aβ-induced neuronal damage. This opens up possibilities for the development of a novel therapeutic approach for the treatment of AD.

## Introduction

Alzheimer's disease (AD) is a highly prevalent neurodegenerative disorder and the leading cause of dementia in the elderly [1]. The characteristic symptoms of AD patients, including progressive cognitive impairment, memory loss, and behavioral deficits, are closely related to pathologic changes in the brain [2]. Senile plaques, a key pathological feature of AD, are essentially composed of the amyloid-beta (Aβ) peptide. Aβ is 39–43 residues long and is generated by two successive proteolytic cleavages of the amyloid precursor protein [3]. AD cases are thought to be chiefly associated with the apparent failures in regulating Aβ production and clearance, leading to increased levels of Aβ and consequent neurotoxicity. Neurotoxic Aβ is initially released as a monomer; molecular interactions then cause it to aggregate into oligomers, fibrils, and plaques in AD brains [4]. The most aggregation-prone form, Aβ_1-42_, which is the predominant and initial species deposited in the brain parenchyma, is considered to be the major pathogenic form in AD [5]. Oligomers are the most toxic Aβ species [6], [7]. However, protofibrillar and fibrillar aggregates including senile plaques are also toxic [8], [9].

Although Aβ aggregation leading to deposition is a critical event in AD [10], the factors that affect Aβ aggregation and accumulation are not completely characterized. It is widely accepted that a considerable number of environmental factors as well as some intrinsic properties of Aβ can work in concert to cause Aβ deposition and aggregation. These factors can influence the thermodynamic stability of the various accessible conformations of Aβ that potentially cause AD. Recent evidence suggests that key subdomains within Aβ affect its propensity toward aggregation. The N-terminal domain of Aβ seems to play an important role in the transition from soluble aggregates to insoluble plaques and acts as a regulatory site controlling both the solubilization and disaggregation process of the Aβ molecule [11], especially the 10 N-terminal residues of Aβ [12]. Intriguingly, site-directed antibodies towards the N-terminal residues 3–6 can relieve amyloid burden in the brain of an AD mouse model and improve their ability to perform cognitive tasks [13]. Meanwhile, a few studies suggest that some other regions of Aβ also play important roles in aggregation, including residues 17–20, 26–30, 30–35, and 39–41 [12], [14], [15], [16]. In terms of therapeutic development, drugs locking these key regions with high specificity can affect the dynamics of the entire Aβ molecule, preventing Aβ self-aggregation and enabling the resolubilization of previously formed aggregates.

Compounds that block Aβ aggregation may ultimately be clinically useful for treating AD [4], [17]. Over the years, much effort has been directed toward screening and designing compounds that inhibit the aggregation and toxicity of Aβ. It is reported that various compounds have inhibitory effects on the aggregation of Aβ, such as Aβ antibodies [18], protease (β- or γ-secretase) inhibitors [19], anti-inflammatory drugs [20], cinnamon extract [21]. However, the stability, safety, validity, cost, and development time limit the suitability of using these agents for different purposes. Recently, peptide-based drugs are now viable alternatives to biopharmaceuticals [22] and are comparable with antibodies in some cases. As drug candidates, peptides have several advantages over antibodies including lower manufacturing costs, higher activity per mass, lower royalty stack, greater stability, and a lower chance of unintended interactions with the immune system [22].

A number of peptides have been designed to bind and inhibit Aβ based on the sequences and structures related to the self-assembling property of Aβ. Some of these peptides not only have especially strong anti-Aβ aggregation effects, but they can also inhibit Aβ neurotoxicity in vitro. More importantly, a few peptides can also reduce cerebral amyloid deposition and attenuate AD-type cognitive deterioration. For example, Austen et al. designed peptide-based aggregation inhibitors containing the binding region (residues 16–20) and retro-inversion of these sequences; these peptides can also inhibit Aβ neurotoxicity in vitro [23], [24], [25]. N-methylation peptides of regions corresponding to the amyloid self-recognition elements (e.g., residues 17–20) can prevent Aβ aggregation and inhibit Aβ-induced toxicity in vitro [26], [27], [28], [29], [30], [31], [32]. Moreover, two N-methylation peptides can reverse the Aβ-induced inhibition of long-term potentiation at remarkably low stoichiometry [32]. Nevertheless, there may be some potential limitations associated with some of these peptide therapies, including insolubility and toxicity [32]. The various β-sheet breaker peptides and the amyloid sequence-derived pentapeptide LPYFDa effectively inhibit fibrillogenesis and the subsequent deposition of amyloids both in vitro and in vivo; furthermore, they can improve behavior in AD model animals [33], [34], [35], [36], [37]. D-amino acid peptides attenuate Aβ aggregation and cell toxicity, and reduce amyloid plaque load in transgenic mice [38], [39], [40]. Although the high dose necessary to observe these effects may preclude their use as a preventive or therapeutic drug [39], they may be suitable for use as probes for detecting amyloid plaques in living brains [38]. However, despite these promising results, very few aggregation inhibitors have reached clinical trials.

Phage display technology is a powerful method for identifying peptides that can target any type of biomolecule [41]. It is a technique in which bacteriophages are engineered to insert a foreign DNA fragment with their capsid proteins and hence express a peptide on their external surfaces. The peptides selected by this method tend to be directed toward biologically relevant sites on the surface of the target protein. Consequently, peptides derived from library screenings often modulate the target protein's activity both in vitro and in vivo and can be used as lead compounds in drug design as well as alternatives to antibodies for target validation in drug discovery [42]. Recently, 20- and 12-mer peptides were used to screen peptides specific to variable lengths or forms of Aβ [12], [38], [43], [44].

As potential therapeutic agents, peptides that directly bind to Aβ are still highly desirable. Although phage display with Aβ_1-10_ as the target to find such peptides is not reported, we reasoned that this method might yield Aβ_1-10_-specific peptides. Here, we successfully identified such a peptide and investigated its properties.

## Materials and Methods

### Biopanning of M13 phage display library against Aβ_1-10_

A randomized 12-mer peptide library presented on M13 phages (PhD-C7C; New England Biolabs, USA) was used for screening against Aβ_1-10_ (Xi'an Huachen Biotechnology, China). The well of an ELISA plate (Shanghai Go On Chemical Ltd., China) was coated with a 100-µl aliquot of 100 µg/ml streptavidin (Sigma, USA) and 0.1 mol/l NaHCO_3_ (pH 8.6) overnight at 4°C. A 10-µl aliquot of 2.0×10^13^ phage units of purified phage from the initial library with 100 µg/ml biotin-Aβ_1-10_ (Xi'an Huachen Biotechnology, China) was solubilized in 400 µl phosphate-buffered saline (PBS) with 0.05% Tween-20 (PBST, pH 7.4). The mixed solution was subsequently incubated for 60 min at 37°C and overnight at 4°C to allow complete binding between phages and Aβ_1-10_. The next day, the solution in the ELISA plate was discarded, and the mixed solution was added to the well for 60 min. The well was washed 5 times with PBST followed by 5 washes with glycine–HCl in order to eliminate the non-specific bound phages. Then, 0.1 mmol/l NHS–LC–Biotin (Sigma, USA) was added to the well and incubated with agitation for 10 min twice. Next, phages bound to the target were eluted with 0.2 mol/l glycine–HCl buffer (pH 2.2) with 0.1% bovine serum albumin (Sigma, USA). After 10 min, the solution was neutralized with 15 µl 1 M Tris–HCl buffer (pH 9.1).

After the round of biopanning, logarithmically growing *Escherichia coli* (ER2738 host strain; New England Biolabs, USA) was infected with a portion of the eluted phages. After amplification, the bacteria were removed by centrifugation, and the phages were purified by serial precipitation with a 20% PEG-8000/2.5 M NaCl solution overnight at 4°C. The phage pellet obtained by centrifugation was finally solubilized in TBS (50 mM Tris-HCl and 150 mM NaCl) supplemented with 0.02% NaN_3_.

The titers of eluted phages were determined by serial dilution after the round of biopanning. Amplification was determined by counting the blue plaques obtained after *E. coli* infection and culturing on a selective medium containing isopropyl-beta-d-thiogalactoside (ICN; Biomedical, Belgium) and 5-bromo-4-chloro-3-indolyl-beta-d-galactopyranoside (Sigma, USA). The remaining phages were grown in bacterial culture overnight, pack-aggregated, expressed through co-infection with a helper phage, and precipitated from the bacterial supernatant. The precipitated phages were then used for subsequent rounds of panning until an obvious enrichment phenomenon appeared.

### Assay to determine the affinity of the selected phages for Aβ_1-10_

Real-time biomolecular interaction analysis (BIA; Amersham Pharmacia, USA) based on surface plasmon resonance was used to assess the affinity of the interaction between the selected phages and Aβ_1-10_. In the binding specificity test, 100 µg/ml streptavidin and 0.1 mmol/l biotin were immobilized in flow cell 1, and 100 µg/ml streptavidin and 100 µg/ml biotin-Aβ_1-10_ in flow cell 2, by amine coupling onto the carboxylated dextran layer of a CM5 chip. The screened phage (1.0×10^11^ phage units) was then injected over flow cells 1 and 2 at 5 µl/min for 6 min. The interaction was monitored as a change in the SPR signal. Greater differences in the response signals (RU) between cells 2 and 1 indicate higher affinity. The procedure of the competitive inhibition test was similar to that of the binding specificity test, except that the selected phage pre-combined with Aβ_1-10_ was injected. BIA evaluation 3.2 software was used to assume one-to-one binding. For the Biacore experiments, samples were run in duplicate, with HBS-EP (5 mM HEPES, 150 mM NaCl, 3.4 mM EDTA, and 0.005% surfactant P20 (pH 7.4) as the running buffer. One surface of the chip in each experimental setup was activated and deactivated, and used as a reference. The response from the reference surface was subtracted from all curves when evaluating the results.

### Sequencing of selected phage clones and synthesis of the special peptide

Phage single-stranded DNA was isolated using phenol/chloroform extraction [45]. The phage genome was sequenced using the Sanger method with two sequencing primers: 5-^HO^GTA TGG GAT TTT GCT AAACAA C-3 and 5-^HO^CCC TCA TAG TTA GCG TAA CG-3 (New England Biolabs, USA). The DNA sequence was analyzed on a CEQ 2000 XL DNA Analysis System (Beckman-Coulter, Belgium). The sequence was read automatically using the JaMBW 1.1 software.

The optimal monoclone phage displaying sequence was selected and synthesized by Xi'an Huachen Biotechnology Company, using standard solid-phase peptide synthesis. All peptides were purified and characterized by MALDI-TOF mass spectrometry.

### Morphological changes in Aβ_1-42_ aggregation under various conditions

Aβ_1-42_ was dissolved in sterile filtered H_2_O at 1.0 mg/ml and was immediately diluted to 0.5 mg/ml (115 µM) in PBS with or without an equimolar concentration of the special peptide or selected phage (1.0×10^11^ phage units), Aβ_1-10_, or Aβ_1-42_ alone as a control. Aggregation was allowed to progress in an incubator for 7 days at 37°C. Electron micrographs were taken using a JEOL 100CX transmission electron microscope with uranyl acetate negative staining.

### Cell toxicity assay

Aβ_1-42_ was dissolved in sterile filtered H_2_O at 100 µM. The solution was incubated in an incubator at 37°C for 7 days, so that Aβ would go into the “aggregated phase,” and stored at 4°C before use. Low-differentiated PC12 cells (Cell Bank, China) were maintained in logarithmic-phase growth on poly-l-lysine-precoated (Sigma, USA) 100-mm dishes (Corning, USA) in DMEM (Gibco, USA) containing 10% fetal bovine serum, 100 units/ml penicillin, and 100 µg/ml streptomycin. Cultures were incubated at 37°C in a 5% CO_2_ humidified atmosphere.

Different concentrations of aggregated Aβ_1-42_ were tested to assess its effect on neuronal cell viability. After PC12 cells were incubated with Aβ_1-42_ (0 (control), 0.01, 0.1, 1, 10, and 20 µM) for 24 h, cell viability was measured using cell counting kit-8 (CCK-8; Beyotime Institute of Biotechnology, China).

To evaluate the role of the special peptide in Aβ_1-42_-mediated neurotoxicity, PC12 cells were incubated in the presence of 20 µM Aβ_1-42_ together with several final concentrations of the special peptide (0.0004, 0.004, 0.02, 0.1, 0.5, and 2.5 µg/µl) for 24 h. To investigate whether the observed effects were due to the special peptide, PC12 cells were treated with the same final concentrations of the special peptide alone for 24 h and cell viability was subsequently evaluated.

The CCK-8 kit was used to count living cells. Briefly, 10 µl of the kit reagent was added to the cells treated as described above in 96-well plates and incubated for 3 h. Cell viability was assessed using the ELISA plate reader at 450 nm.

### Apoptosis assessment with propidium iodide staining

Propidium iodide (PI) staining was performed to evaluate the status of apoptosis as follows. PC12 cells were incubated for 24 h with any one of the following three treatments: (1) without the special peptide or aggregated Aβ_1-42_ as a control, (2) with 20 µM Aβ_1-42_ only, and (3) with both 20 µM Aβ_1-42_ and the special peptide at concentrations of 0.0004, 0.004, 0.02, 0.1, 0.5, and 2.5 µg/µl. All samples were centrifuged for 10 min at 800×*g*, and the supernatants were discarded. After washing twice with PBS, the supernatants were fixed in 70% ethanol (100 µl) overnight at –20°C. The cells were then centrifuged for 10 min at 1,200×*g*, washed twice with PBS, and stained with the DNA-specific fluorochrome propidium iodide (PI; Sigma, USA) at a terminal concentration of 50 µg/µl. The mixed cells were then filtered and incubated in the dark at 4°C for 30 min before flow cytometric analysis (Beckman-Coulter, USA). A total of 1,000 cells were counted to determine the percentage of cells exhibiting the morphological hallmarks of apoptosis such as DNA fragmentation, nuclear condensation, or segmentation.

### Animals, surgery, and drug administration

Ethics statement: this study was approved by the Committee on the Ethics of Animal Experiments of Anhui Medical University (Permit Number: 12-2866). All animal procedures were carried out in strict accordance with the recommendations in the Guide for the Care and Use of Laboratory Animals of the National Institutes of Health. All surgery was performed under chloral hydrate, and all efforts were made to minimize suffering.

Forty experimentally naive male Sprague–Dawley rats (SD; Beijing Vital River Experimental Animal Technology, China), weighing 260–280 g at the beginning of the experiment were used. They were housed individually in a room maintained at 23°C with a 12-h light–dark cycle (lights on at 08:00 h) for the duration of the experiment. Rats were allowed free access to food and water except during experimental testing. Rats anesthetized with chloral hydrate (350 mg/kg, i.p.) were positioned in a stereotaxic instrument (RWD Life Science, China), and a cannula (RWD Life Science, China) was implanted into the left cerebral ventricle (A: 0.3 mm, L: 1.2 mm, V: 3.6). Rats were allowed 5 days of recovery after the surgery. The correct location of the cannula was checked by dissecting the brain following the completion of the experiments. Only animals with correctly placed cannulas were used in the evaluation of the experiments. Six rats in each group were used for the final analysis. The special peptide was dissolved in 0.9% saline solution before use. Aβ_1-42_ (Sigma, USA) was also dissolved in 0.9% saline solution. The solution was then incubated at 37°C for 7 days to induce formation of aggregated Aβ and stored at 4°C before use. For intracerebroventricular administration in vivo, aggregated Aβ_1-42_, the special peptide binding to the Aβ_1-10_, or saline solution was infused with the aid of a mini-pump (RWD Life Science, China). The rats were randomly divided into three groups. (1) AD model group: the AD model rats were established as described previously [46]. After surgery, the rats were injected with 3 µl aggregated Aβ_1-42_ (1 µg/µl, 0.8 µl/min) on days 5, 8, and 11, and with 3 µl saline solution (0.6 µl/min) on the other days, days 5–14 (2) The special peptide treatment group with three subgroups: AD model rats were infused daily with 3 µl special peptide at different final concentrations (0.110, 0.522, and 2.610 µg/µl at 0.6 µl/min) for 14 consecutive days from day 5 onwards. (3) Control group: the rats were infused daily with 3 µl saline solution (0.6 µl/min) alone throughout the same period. Upon completion of behavioral testing, the rats were sacrificed by decapitation.

### Morris water maze task

The apparatus and procedure were slightly modified as described previously [47]. Briefly, the water maze apparatus consisted of a circular pool 150 cm in diameter, 60 cm deep, and filled daily to a height of 30 cm with fresh tap water (21∼22°C). A black escape platform (10 cm diameter, 24 cm height) was placed in one of the 4 quadrants of the pool (in target quadrant 2) and submerged approximately 1.5 cm below the water surface. Each rat's swimming performance was monitored by a video camera linked to a computer-based image analyzer (Chinese Academy of Medical Sciences, China). Place learning was tested for 10 consecutive days. Each rat was trained to find the platform with 4 successive trials a day. The sequence of water-entering points differed each day, but the location of the platform was constant. The time taken to find the platform (escape latency), swimming speed, and the distance traveled were measured and averaged over 4 trials. If a rat failed to find the platform within 60 s, it was placed on the platform manually; regardless of whether the rat found the platform, it was kept there for 30 s. Each animal was subsequently returned to its cage until the next trial. After all of the rats completed 1 trial, the next trial began and followed the same order of rats. On day 10 of training, an additional trial was given as a probe trial by removing the platform. The animals were placed in the quadrant where the platform was previously located in the fourth starting position and were put through a single test-free swimming period for 60 s. The percentage of distance and time spent in the quadrant where the platform was previously located (target quadrant) were used as measures of spatial memory.

### Statistical analysis

The data followed a normal distribution, and are presented as the mean ± standard error of the mean (SEM). For the place learning test in the Morris water maze task (MWM task), analysis was performed using a two-way repeated-measures analysis of variance (ANOVA) with day and treatment as independent variables. For the other tests, statistical analysis was performed using ANOVA with groups as the independent variable. Post-hoc analysis using Tukey test was used to compare results for different days and groups. Statistical significance was set at *P*<0.05.

## Results

### Selection of specific phages binding to Aβ_1-10_

After three rounds of screening, the eluted phage titers progressively increased after each round of panning (*Ps*<0.01), suggesting that non-specific phages were eluted and specific phages were amplified.

### Affinity of Aβ_1-10_ for the selected phages

**Figure 1:**
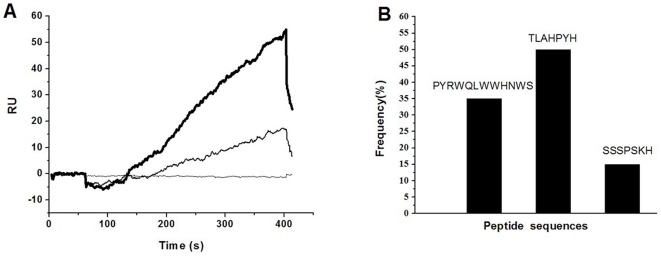
The specific affinity and amino acid sequences of the selected phages. (A) Specific affinity between Aβ_1-10_ and the phages screened for three rounds. Real dark line: binding specificity test of the screened phages; Dash line: competitive inhibition test of the small peptide; Horizon line: the baseline of reaction unit; (B) Amino acid sequence of peptides displayed by the 20 selected phage clones. Frequency of peptides was displayed by the phage clones. Three sequences were highly represented.

In the binding specificity test, as reaction time increased, the positive difference of RU between flow cells 2 and 1 gradually increased (Fig. 1A), indicating that the combined power of the selected phages and streptavidin–biotin–Aβ_1-10_ was significantly stronger than that of the selected phages and streptavidin–biotin. This result suggests that high binding specificity and affinity exist between the selected phages and Aβ_1-10_. In the competitive inhibition test, as reaction time increased, the positive difference in RU between flow cells 2 and 1 also gradually increased (Fig. 1A). This result indicates that the bonding force between the selected phages pre-combined with Aβ_1-10_ and streptavidin–biotin–Aβ_1-10_ in cell 2 was also higher than that between the selected phages and streptavidin–biotin in flow cell 1. However, the positive difference between cells 2 and 1 in the competitive inhibition test was lower than that in the binding specificity test for all corresponding time points; this is because the binding site of the selected phages is partly locked by the pre-combined Aβ_1-10_. Together, the results suggest that the affinity between the selected phages and Aβ_1-10_ is highly specific.

### Sequencing of the selected phage clones and synthesis of the special peptide

The DNA of the 20 clones selected on the basis of their affinity for Aβ_1-10_ was sequenced (Fig. 1B). The library was enriched in three sequences: PYRWQLWWHNWS, TLAHPYH, and SSSPSKH. Since the 12-mer peptide library was used for screening against Aβ_1-10_, TLAHPYH and SSSPSKH were regarded as contaminants of the other sequences selected in our experiment. The target clone possessed a peptide, PYRWQLWWHNWS, which was then used to synthesize the special peptide.

### Morphological changes in Aβ_1-42_ aggregation under various conditions

**Figure 2:**
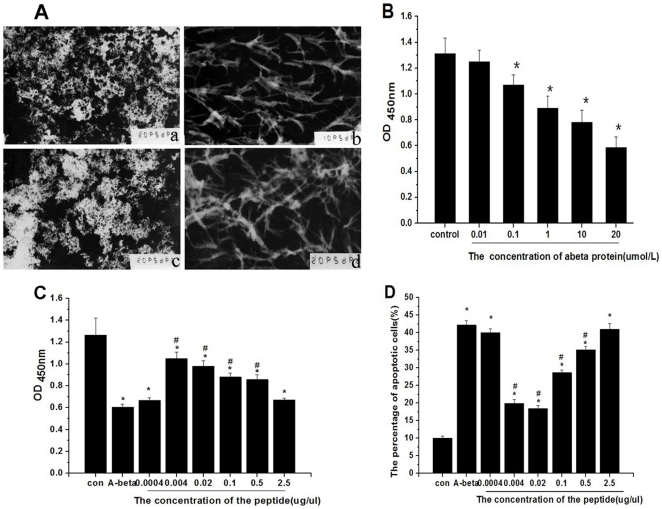
The special peptide alleviates Aβ aggregation and toxic effects *in vitro*. (A) Electron microscopic analysis of morphological change showing the effect of the special peptide or selected phage on Aβ_1-42_ aggregation. Aβ_1-42_ (a) or Aβ_1-10_ (c) alone was incubated for 7 days; Aβ_1-42_ with special peptide (b) or the selected phage (d) was incubated for 7 days; (B) Different concentrations of aggregated Aβ_1-42_ were tested to assess its effect on neuronal cell viability by CCK-8 assay. *P<0.05 when compared with the control group; (C) Cell viability was assessed in the presence of 20 µM Aβ_1-42_ and different concentrations of the special peptide by CCK-8 assay. *P<0.05 when compared with the control group. ^#^ *P*<0.05 when compared with the group treated with 20 µM Aβ_1-42_ only (note: 0.04 µg/µl peptide equal to 20 µM); (D) Detection of apoptosis in PC12 cells by PI staining. *P<0.01 when compared with the control group. *^#^P*<0.01 when compared with the group treated with 20 µM Aβ_1-42_ only.

Some obvious plaques were observed for Aβ_1-42_ or Aβ_1-10_ alone. However, after the addition of the selected phages or the special synthetic peptide, the plaques were instead of bundles of short fibrils. This indicates that the selected phages and special synthetic peptide can inhibit the aggregation of Aβ_1-42_ into plaques (Fig. 2A).

### Effects of the special peptide on Aβ_1-42_-mediated neurotoxicity *in vitro*

As shown in Fig. 2B, the viability of cells treated with Aβ_1-42_ only at concentrations >0.01 µM was significantly decreased in a concentration-dependent manner (*Ps*<0.05). Based on these results, a final concentration of 20 µM Aβ_1-42_ was selected as the optimal concentration for subsequent experiments because cell viability was about 60% at that concentration. In addition, cell death with 20 µM Aβ_1-42_ appeared to be mainly due to apoptosis (Fig. 2D).

To investigate the effect of the special peptide on Aβ_1-42_-medidated neurotoxicity, PC12 cells were incubated in the presence of 20 µM Aβ_1-42_ together with several concentrations of the special peptide. Compared with the control, cell viability was significantly decreased with 20 µM Aβ_1-42_ treatment (*P*<0.01). In the treatment groups, the pattern of cell viability exhibited an inverted “U” shape. The intermediate concentrations (i.e., 0.004, 0.02, 0.1, and 0.5 µg/µl) of the special peptide significantly increased cell viability (*Ps*<0.05), while smaller (0.0004 µg/µl) and larger doses (2.5 µg/µl) were ineffective (*Ps*>0.05) (Fig. 2C). This neuroprotective effect of the special peptide on Aβ_1-42_-induced cell death resulted completely from the net action of the special peptide on Aβ_1-42_ aggregation, because the viabilities of PC12 cells incubated with different concentrations of the special peptide alone were similar to those of the control group (data not shown).

PI staining revealed that the percentage of apoptotic cells among groups exhibited a “U” shape; i.e., the percentage of apoptotic cells was significantly higher in the presence of 20 µM Aβ_1-42_ than that of the control (*P*<0.05). Furthermore, the increase was markedly attenuated by combined treatment with several concentrations of the special peptide (i.e., 0.004, 0.02, 0.1, and 0.5 µg/µl) (*Ps*<0.05), but it was not significantly different from these doses (*Ps*>0.05) (Fig. 2D).

### Performance in the MWM test

**Figure 3:**
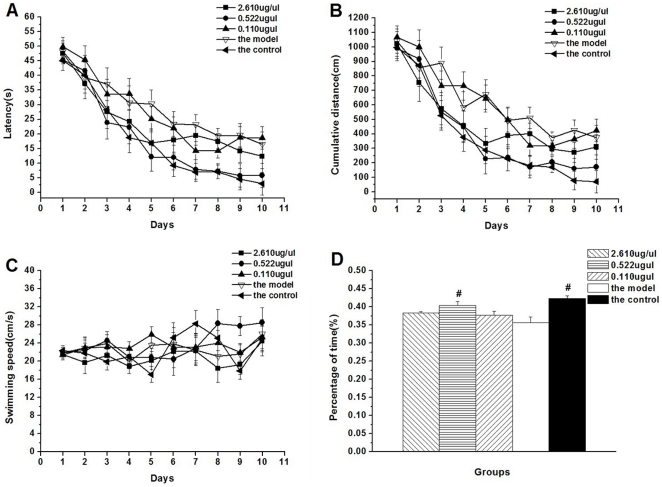
Performance of the rats in the Morris water maze. (A) latency, (B) distance and (C) swimming speed in the place learning test; (D) the percentage of time spent in the target quadrant in the probe trial. *^#^P*<0.05 when compared with the model group. (note: the 0.522 µg/µl special peptide equimolar to Aβ_1-42_).

Fig. 33 shows the performance of rats in finding the hidden platform in the MWM task. The latency progressively decreased with days for all rats combined (*F*_(9,225)_  = 91.49, *P*<0.01; Fig. 3A), suggesting that the rats were able to learn the task. The repeated-measures ANOVA indicates that the effect of treatment on the latency was significant (*F*_(4,25)_  = 3.121, *P* = 0.0324). Post-hoc analysis shows that the latency in the AD model rats was longer than that in the control rats (*P* = 0.041). However, among the three special peptide-treated groups, only the latency of the 0.522 µg/µl group was significantly shorter than that of the AD model group (*P* = 0.047), and was similar to that of the control group.

The cumulative distance also declined daily (*F*_(9,225)_  = 78.09, *P*<0.01; Fig. 3B). The repeated-measures ANOVA indicates that treatment had significant effect on the cumulative distance (*F*_(4,25)_  = 4.941, *P* = 0.005). The AD model rats swam significantly longer distances than the control rats (*P* = 0.021); only the 0.522 µg/µl special peptide-treated group had a shorter latency than the AD model group (*P* = 0.029). The results of the latency and distance suggest that repeated daily infusion of the special peptide at 0.522 µg/µl significantly ameliorates the impairment of performance caused by Aβ_1-42_. Swimming speed was similar among groups (*F*_(4,25)_  = 0.897, *P* = 0.467; Fig. 3C), indicating that the cognitive performance observed under the various treatments is not due to potential alterations of locomotor function.

In the probe trial test, the percentage of time in the target quadrant showed significant effect of treatment (*F*_(4,25)_  = 7.984, *P*<0.01; Fig. 3D). This indicates that the AD model rats searched the target quadrant for a significantly less time than the control rats (*P*<0.001); only the 0.522 µg/µl special peptide-treated rats spent significantly more time than the AD model rats (*P* = 0.025). The percentages of cumulative distance in each group of rats corresponded with the percentages of escape latency (data not shown).

## Discussion

Using phage display technology, we identified a peptide that binds specifically to Aβ_1-10_. Meanwhile, the special peptide inhibited the aggregation of Aβ_1-42_ into plaques, and reduced neuronal damage induced by Aβ_1-42_ in PC12 cells at appropriate concentrations. Moreover, the special peptide exhibited a protective effect against Aβ_1-42_-induced learning and memory deficits in the MWM task.

Based on previous studies, we reasoned that a peptide could be selected by phage display technology using Aβ_1-10_ as the target. After three rounds of screening, the phages were selected. The affinity between the selected phages and Aβ_1-10_ was assessed by BIA; the results show that the affinity between them was highly specific. Meanwhile, we found that this method is suitable for identifying positive correlations between selected phages and peptides; it is superior to the orthodox ELISA because it maintains the natural properties of biomolecules without the need for fluorescent and isotope labeling, therefore making the results more reliable. Three amino acid sequences were identified; the sequence of one was interesting, while the others were considered to be contaminants. We chose to use this target sequence to synthesize the special peptide.

The presence of the selected phages or special peptide elicited dramatic morphological features in that some observed plaques were instead of bundles of fibrils. This suggests that specific phages and the special peptide could inhibit Aβ aggregation into plaques. Although this method led to fibril formation, which is considered to be an aggregation pathway originating from a high entropic barrier and a thermodynamically unfavorable event, a recent study indicates that the equilibrium between toxic and non-toxic Aβ intermediates exhibits a dynamic nature [48]. Furthermore, the non-aggregated and aggregated states of Aβ are in equilibrium, and soluble forms are more accessible to clearance and degradation than insoluble forms [49]. In this study, the special peptide was able to disaggregate plaques due to steady-state equilibrium between Aβ in plaques and in the monomeric form [50]. This ultimately led to increased amounts of monomeric Aβ, which is more easily cleared from the brain [51] and can play a neuroprotective role in the brain [52].

Extracellular Aβ peptides are highly cytotoxic to neuronal cells, and the underlying mechanisms may include free radical damage, oxidative stress, and mitochondrial dysfunction of neurons, which ultimately induce apoptosis [2]. Through interactions with unidentified targets on the cell surface, Aβ initiates a cascade of intracellular events that culminate in neuronal death. Recent studies show that the dying cells exhibit apoptotic characteristics in AD brains and cultures of primary neurons and neuronal cell lines exposed to Aβ [53], [54]. Furthermore, apoptosis in neurons may be responsible for neuronal death in AD [55]. Applying Aβ to cultured cells even at micromolar concentrations can cause apoptosis [56]. PC12 cells were used in this study as a neuronal model because they biochemically and morphologically resemble neurons after differentiation and are particularly sensitive to Aβ peptides [57]. Moreover, they are relatively easy to culture and survive longer than primary cultured neurons [58]. We found that Aβ-induced cell death was attenuated by the addition of the special peptide at 0.004, 0.02, 0.1, and 0.5 µg/µl. Meanwhile, the percentage of apoptotic cells was markedly reduced by combined treatment with the special peptide (i.e., 0.004, 0.02, 0.1, and 0.5 µg/ml). The positive effect of the special peptide was significant at a molar ratio of peptide: Aβ ranging from 0.1 to 12.5. Interestingly, the relationship between changes in concentration and efficacy of the special peptide in these experiments met the pattern of inverted U-shaped dose-effect curve (IUSDEC). The IUSDEC is widely described and poorly understood phenomenon in the pharmacological field. The basic concept is that, the effects of increasing dosages of a given compound appear to increase up to a maximum, and then the effects decrease [59]. A recent comprehensive review has exhibited many examples of such curves in pharmacology, such as cardiac glycosides, anti-tumor drugs and drugs of central nervous system, chemoprevention of stroke, traumatic brain injuries [60]. Similarly, it is difficult to elucidate the mechanisms underlying the IUSDEC in our study. We speculated that the lower concentration of the special peptide might be too low to interfere sufficiently the interaction between the special peptide and Aβ, so that the protective effect could not be produced. On the other hand, the reduced effect at the higher concentration of the special peptide might be due to that it stimulates the body to generate endogenous Aβ or enhances the toxic process of Aβ, or produces self-cytotoxicity. For the latter, however, our unpublished data had suggested that the special peptide had no cytotoxicity for PC12 cells, even up to a concentration of 2.5 µg/µl.

An animal AD model induced by intracerebroventricular Aβ infusion is particularly attractive for evaluating drugs for AD. Ample experimental evidence indicates that single or chronic intracerebroventricular administration of several kinds of Aβ peptides (i.e., Aβ_25-35_, Aβ_1-40_, and Aβ_1-42_) can induce cognitive impairment [61], [62], [63], [64]. Although the effects of Aβ on learning and memory have been extensively studied, the mechanism by which Aβ causes cognitive deficits is not clearly understood. A recent study suggests that the spatial learning and memory deficits induced by Aβ peptides in rodents may not be entirely related to Aβ-induced neuronal damage such as the activation of glial cells, and neuroinflammatory and oxidative responses [65]. And it is also indicated that Aβ_1-42_-induced mitochondrial mislocalization contributes to late-onset behavioral deficits in a transgenic Drosophila model [66]. According to the validated in vitro concentrations of the special peptide, we chose three in vivo doses (i.e., 0.110, 0.552, and 2.610 µg/µl); the corresponding molar ratios of peptide: Aβ were 1, 5, and 25, respectively. The results showed that Aβ_1–42_ injection into the lateral ventricle induced spatial learning and memory impairment, and infusing the special peptide alone at 0.552 µg/µl significantly ameliorated impairment in the MWM. The beneficial effect of the special peptide on learning and memory may be related to its activity as a direct binder to Aβ_1–10_. Further studies are needed to fully understand the mechanisms of the protective activity of the peptide against the Aβ_1–42_-induced learning and memory impairment. Note should be made that a relatively small difference occurred among groups in the MWM, which might be attributable to the AD model based on intracerebroventricular infusion of Aβ or/and the inadequate sample size in this study. Further, the effect of the special peptide on cognitive function should be confirmed using AD transgenic mice, such as the 5xFADAD model mice [21].

In summary, by using phage display technology, we successfully identified a 12-amino acid peptide that can specifically bind to Aβ_1-10_. Such a peptide may not only serve to inhibit the aggregation of Aβ into plaques, but also inhibit the toxic effects of Aβ. Therefore, the special peptide may be a potential drug for the treatment of AD.

## References

[1] Ferri CP, Prince M, Brayne C, Brodaty H, Fratiglioni L (2005). Global prevalence of dementia: a Delphi consensus study.. Lancet.

[2] Hardy J, Selkoe DJ (2002). The amyloid hypothesis of Alzheimer's disease: progress and problems on the road to therapeutics.. Science.

[3] Selkoe DJ (1996). Amyloid beta-protein and the genetics of Alzheimer's disease.. J Biol Chem.

[4] Esler WP, Stimson ER, Jennings JM, Vinters HV, Ghilardi JR (2000). Alzheimer's disease amyloid propagation by a template-dependent dock-lock mechanism.. Biochemistry.

[5] Crews L, Masliah E (2010). Molecular mechanisms of neurodegeneration in Alzheimer's disease.. Hum Mol Genet.

[6] Findeis MA (2007). The role of amyloid beta peptide 42 in Alzheimer's disease.. Pharmacol Ther.

[7] Haass C, Selkoe DJ (2007). Soluble protein oligomers in neurodegeneration: lessons from the Alzheimer's amyloid beta-peptide.. Nat Rev Mol Cell Biol.

[8] Glabe CG (2008). Structural classification of toxic amyloid oligomers.. J Biol Chem.

[9] Crouch PJ, Harding SM, White AR, Camakaris J, Bush AI (2008). Mechanisms of A beta mediated neurodegeneration in Alzheimer's disease.. Int J Biochem Cell Biol.

[10] Bharadwaj PR, Dubey AK, Masters CL, Martins RN, Macreadie IG (2009). Abeta aggregation and possible implications in Alzheimer's disease pathogenesis.. J Cell Mol Med.

[11] Qahwash I, Weiland KL, Lu Y, Sarver RW, Kletzien RF (2003). Identification of a mutant amyloid peptide that predominantly forms neurotoxic protofibrillar aggregates.. J Biol Chem.

[12] Orner BP, Liu L, Murphy RM, Kiessling LL (2006). Phage display affords peptides that modulate beta-amyloid aggregation.. J Am Chem Soc.

[13] Solomon B (2007). Beta-amyloidbased immunotherapy as a treatment of Alzheimers disease.. Drugs Today (Barc).

[14] Nelson TJ, Alkon DL (2007). Protection against beta-amyloid-induced apoptosis by peptides interacting with beta-amyloid.. J Biol Chem.

[15] Liu R, McAllister C, Lyubchenko Y, Sierks MR (2004). Residues 17-20 and 30-35 of beta-amyloid play critical roles in aggregation.. J Neurosci Res.

[16] Morimoto A, Irie K, Murakami K, Masuda Y, Ohigashi H (2004). Analysis of the secondary structure of beta-amyloid (Abeta42) fibrils by systematic proline replacement.. J Biol Chem.

[17] Cummings JL, Doody R, Clark C (2007). Disease-modifying therapies for Alzheimer disease: challenges to early intervention.. Neurology.

[18] Schenk D (2004). Hopes remain for an Alzheimer's vaccine.. Nature.

[19] Frisardi V, Solfrizzi V, Imbimbo PB, Capurso C, D'Introno A (2010). Towards disease-modifying treatment of Alzheimer's disease: drugs targeting beta-amyloid.. Curr Alzheimer Res.

[20] Thomas T, Nadackal TG, Thomas K (2001). Aspirin and non-steroidal anti-inflammatory drugs inhibit amyloid-beta aggregation.. Neuroreport.

[21] Frydman-Marom A, Levin A, Farfara D, Benromano T, Scherzer-Attali R (2011). Orally administrated cinnamon extract reduces beta-amyloid oligomerization and corrects cognitive impairment in Alzheimer's disease animal models.. PLoS One.

[22] Ladner RC, Sato AK, Gorzelany J, de Souza M (2004). Phage display-derived peptides as therapeutic alternatives to antibodies.. Drug Discov Today.

[23] Austen BM, Paleologou KE, Ali SA, Qureshi MM, Allsop D (2008). Designing peptide inhibitors for oligomerization and toxicity of Alzheimer's beta-amyloid peptide.. Biochemistry.

[24] Matharu B, El-Agnaf O, Razvi A, Austen BM (2010). Development of retro-inverso peptides as anti-aggregation drugs for beta-amyloid in Alzheimer's disease.. Peptides.

[25] Taylor M, Moore S, Mayes J, Parkin E, Beeg M (2010). Development of a proteolytically stable retro-inverso peptide inhibitor of beta-amyloid oligomerization as a potential novel treatment for Alzheimer's disease.. Biochemistry.

[26] Hughes E, Burke RM, Doig AJ (2000). Inhibition of toxicity in the beta-amyloid peptide fragment beta -(25-35) using N-methylated derivatives: a general strategy to prevent amyloid formation.. J Biol Chem.

[27] Sciarretta KL, Boire A, Gordon DJ, Meredith SC (2006). Spatial separation of beta-sheet domains of beta-amyloid: disruption of each beta-sheet by N-methyl amino acids.. Biochemistry.

[28] Gordon DJ, Tappe R, Meredith SC (2002). Design and characterization of a membrane permeable N-methyl amino acid-containing peptide that inhibits Abeta1-40 fibrillogenesis.. J Pept Res.

[29] Cruz M, Tusell JM, Grillo-Bosch D, Albericio F, Serratosa J (2004). Inhibition of beta-amyloid toxicity by short peptides containing N-methyl amino acids.. J Pept Res.

[30] Kokkoni N, Stott K, Amijee H, Mason JM, Doig AJ (2006). N-Methylated peptide inhibitors of beta-amyloid aggregation and toxicity. Optimization of the inhibitor structure.. Biochemistry.

[31] Findeis MA (2002). Peptide inhibitors of beta amyloid aggregation.. Curr Top Med Chem.

[32] Amijee H, Madine J, Middleton DA, Doig AJ (2009). Inhibitors of protein aggregation and toxicity.. Biochem Soc Trans.

[33] Hatip FF, Hatip-Al-Khatib I, Matsunaga Y, Suenaga M, Sen N (2010). Effects of 8-residue beta sheet breaker peptides on aged Abeta40-induced memory impairment and Abeta40 expression in rat brain and serum following intraamygdaloid injection.. Curr Alzheimer Res.

[34] Soto C, Sigurdsson EM, Morelli L, Kumar RA, Castano EM (1998). Beta-sheet breaker peptides inhibit fibrillogenesis in a rat brain model of amyloidosis: implications for Alzheimer's therapy.. Nat Med.

[35] Chacon MA, Barria MI, Soto C, Inestrosa NC (2004). Beta-sheet breaker peptide prevents Abeta-induced spatial memory impairments with partial reduction of amyloid deposits.. Mol Psychiatry.

[36] Tjernberg LO, Lilliehook C, Callaway DJ, Naslund J, Hahne S (1997). Controlling amyloid beta-peptide fibril formation with protease-stable ligands.. J Biol Chem.

[37] Granic I, Masman MF, Kees Mulder C, Nijholt IM, Naude PJ (2010). LPYFDa neutralizes amyloid-beta-induced memory impairment and toxicity.. J Alzheimers Dis.

[38] Wiesehan K, Buder K, Linke RP, Patt S, Stoldt M (2003). Selection of D-amino-acid peptides that bind to Alzheimer's disease amyloid peptide abeta1-42 by mirror image phage display.. Chembiochem.

[39] Wiesehan K, Stohr J, Nagel-Steger L, van Groen T, Riesner D (2008). Inhibition of cytotoxicity and amyloid fibril formation by a D-amino acid peptide that specifically binds to Alzheimer's disease amyloid peptide.. Protein Eng Des Sel.

[40] van Groen T, Wiesehan K, Funke SA, Kadish I, Nagel-Steger L (2008). Reduction of Alzheimer's disease amyloid plaque load in transgenic mice by D3, A D-enantiomeric peptide identified by mirror image phage display.. ChemMedChem.

[41] Zwick MB, Shen J, Scott JK (1998). Phage-displayed peptide libraries.. Curr Opin Biotechnol.

[42] Molek P, Strukelj B, Bratkovic T (2011). Peptide phage display as a tool for drug discovery: targeting membrane receptors.. Molecules.

[43] Kang CK, Jayasinha V, Martin PT (2003). Identification of peptides that specifically bind Abeta1-40 amyloid in vitro and amyloid plaques in Alzheimer's disease brain using phage display.. Neurobiol Dis.

[44] Larbanoix L, Burtea C, Laurent S, Van Leuven F, Toubeau G (2010). Potential amyloid plaque-specific peptides for the diagnosis of Alzheimer's disease.. Neurobiol Aging.

[45] Sambrook J, Gething MJ (1989). Protein structure. Chaperones, paperones.. Nature.

[46] Wang R, Zhang HY, Tang XC (2001). Huperzine A attenuates cognitive dysfunction and neuronal degeneration caused by beta-amyloid protein-(1-40) in rat.. Eur J Pharmacol.

[47] Chen GH, Wang YJ, Qin S, Yang QG, Zhou JN (2007). Age-related spatial cognitive impairment is correlated with increase of synaptotagmin 1 in dorsal hippocampus in SAMP8 mice.. Neurobiol Aging.

[48] Kuperstein I, Broersen K, Benilova I, Rozenski J, Jonckheere W (2010). Neurotoxicity of Alzheimer's disease Abeta peptides is induced by small changes in the Abeta42 to Abeta40 ratio.. Embo J.

[49] Aguzzi A, O'Connor T (2010). Protein aggregation diseases: pathogenicity and therapeutic perspectives.. Nat Rev Drug Discov.

[50] Maggio JE, Stimson ER, Ghilardi JR, Allen CJ, Dahl CE (1992). Reversible in vitro growth of Alzheimer disease beta-amyloid plaques by deposition of labeled amyloid peptide.. Proc Natl Acad Sci U S A.

[51] Tanzi RE, Moir RD, Wagner SL (2004). Clearance of Alzheimer's Abeta peptide: the many roads to perdition.. Neuron.

[52] Giuffrida ML, Caraci F, Pignataro B, Cataldo S, De Bona P (2009). Beta-amyloid monomers are neuroprotective.. J Neurosci.

[53] Majd S, Zarifkar A, Rastegar K, Takhshid MA (2008). Different fibrillar Abeta 1-42 concentrations induce adult hippocampal neurons to reenter various phases of the cell cycle.. Brain Res.

[54] Abdul HM, Calabrese V, Calvani M, Butterfield DA (2006). Acetyl-L-carnitine-induced up-regulation of heat shock proteins protects cortical neurons against amyloid-beta peptide 1-42-mediated oxidative stress and neurotoxicity: implications for Alzheimer's disease.. J Neurosci Res.

[55] Martin D, Salinas M, Lopez-Valdaliso R, Serrano E, Recuero M (2001). Effect of the Alzheimer amyloid fragment Abeta(25-35) on Akt/PKB kinase and survival of PC12 cells.. J Neurochem.

[56] Jellinger KA (2006). Challenges in neuronal apoptosis.. Curr Alzheimer Res.

[57] Harper JD, Lansbury PT (1997). Models of amyloid seeding in Alzheimer's disease and scrapie: mechanistic truths and physiological consequences of the time-dependent solubility of amyloid proteins.. Annu Rev Biochem.

[58] Tan J, Town T, Placzek A, Kundtz A, Yu H (1999). Bcl-X(L) inhibits apoptosis and necrosis produced by Alzheimer's beta-amyloid1-40 peptide in PC12 cells.. Neurosci Lett.

[59] Baldi E, Bucherelli C (2005). The inverted “u-shaped” dose-effect relationships in learning and memory: modulation of arousal and consolidation.. Nonlinearity Biol Toxicol Med.

[60] Calabrese EJ (2008). Hormesis and medicine.. Br J Clin Pharmacol.

[61] Yamada M, Chiba T, Sasabe J, Nawa M, Tajima H (2005). Implanted cannula-mediated repetitive administration of Abeta25-35 into the mouse cerebral ventricle effectively impairs spatial working memory.. Behav Brain Res.

[62] Olariu A, Yamada K, Mamiya T, Hefco V, Nabeshima T (2002). Memory impairment induced by chronic intracerebroventricular infusion of beta-amyloid (1-40) involves downregulation of protein kinase C. Brain Res.

[63] Shin EJ, Nabeshima T, Lee PH, Kim WK, Ko KH (2004). Dimemorfan prevents seizures induced by the L-type calcium channel activator BAY k-8644 in mice.. Behav Brain Res.

[64] Youssef I, Florent-Bechard S, Malaplate-Armand C, Koziel V, Bihain B (2008). N-truncated amyloid-beta oligomers induce learning impairment and neuronal apoptosis.. Neurobiol Aging.

[65] Piermartiri TC, Figueiredo CP, Rial D, Duarte FS, Bezerra SC (2010). Atorvastatin prevents hippocampal cell death, neuroinflammation and oxidative stress following amyloid-beta(1-40) administration in mice: evidence for dissociation between cognitive deficits and neuronal damage.. Exp Neurol.

[66] Iijima-Ando K, Hearn SA, Shenton C, Gatt A, Zhao L (2009). Mitochondrial mislocalization underlies Abeta42-induced neuronal dysfunction in a Drosophila model of Alzheimer's disease.. PLoS One.

